# Benthic infaunal community structuring in an acidified tropical estuarine system

**DOI:** 10.1186/2046-9063-10-11

**Published:** 2014-11-06

**Authors:** M Belal Hossain, David J Marshall

**Affiliations:** 1 Environmental and Life Sciences, Faculty of Science, Universiti Brunei Darussalam, BE1410 Jalan Tunkgu Link, Brunei Darussalam; 2 Department of Fisheries and Marine Science, Noakhali Science and Technology University, Sonapur 3814, Bangladesh

**Keywords:** Community structure, Infauna, Soft-bottom, Tropical estuary, Salinity, Acidification

## Abstract

**Background:**

Recent studies suggest that increasing ocean acidification (OA) should have strong direct and indirect influences on marine invertebrates. While most theory and application for OA is based on relatively physically-stable oceanic ecological systems, less is known about the effects of acidification on nearshore and estuarine systems. Here, we investigated the structuring of a benthic infaunal community in a tropical estuarine system, along a steep salinity and pH gradient, arising largely from acid-sulphate groundwater inflows (Sungai Brunei Estuary, Borneo, July 2011- June 2012).

**Results:**

Preliminary data indicate that sediment pore-water salinity (range: 8.07 - 29.6 psu) declined towards the mainland in correspondence with the above-sediment estuarine water salinity (range: 3.58 – 31.2 psu), whereas the pore-water pH (range: 6.47- 7.72) was generally lower and less variable than the estuarine water pH (range: 5.78- 8.3), along the estuary. Of the thirty six species (taxa) recorded, the polychaetes *Neanthes* sp., *Onuphis conchylega,* Nereididae sp. and the amphipod Corophiidae sp., were numerically dominant. Calcified microcrustaceans (e.g., Cyclopoida sp. and Corophiidae sp.) were abundant at all stations and there was no clear distinction in distribution pattern along the estuarine between calcified and non-calcified groups. Species richness increased seawards, though abundance (density) showed no distinct directional trend. Diversity indices were generally positively correlated (Spearman’s rank correlation) with salinity and pH (p <0.05) and negatively with clay and organic matter, except for evenness values (p >0.05). Three faunistic assemblages were distinguished: (1) nereid-cyclopoid-sabellid, (2) corophiid-capitellid and (3) onuphid- nereid-capitellid. These respectively associated with lower salinity/pH and a muddy bottom, low salinity/pH and a sandy bottom, and high salinity/pH and a sandy bottom. However, CCA suggested that species distribution and community structuring is more strongly influenced by sediment particle characteristics than by the chemical properties of the water (pH and salinity).

**Conclusions:**

Infaunal estuarine communities, which are typically adapted to survive relatively acidic conditions, may be **less exposed, less sensitive, and less vulnerable** than epibenthic or pelagic communities to further acidification of above-sediment waters. These data question the extent to which all marine infaunal communities, including oceanic communities, are likely to be affected by future global CO_2_-driven acidification.

## Background

Many tropical and subtropical estuaries experience acidification (low pH) resulting from acid sulfate soil (ASS) inflows [1-5]. ASS perturbation occurs when pyrite is produced during bacterial breakdown of organic matter in the presence of sulfate and iron oxides in sediments [1]. Though the estuarine pH levels generally vary from 7.0 to 7.5 in the fresher sections, to between 8.0 and 8.6 in the more saline areas [6]; the runoff from the ASS can reduce the pH of adjacent estuaries to as low as 2 [7]. Many studies have demonstrated that aquatic organisms have trouble surviving if pH levels drop under 5 or rise above 9 [7-9]. Fluctuating pH may compromise optimally related life processes (such as metabolism and growth), but also potentially increase the solubility of calcium carbonates and the bioavailability of metals [8,10]. In the acidic condition, toxic metals in the estuarine sediment can be resuspended in the water columns which have indirect detrimental impacts on many organisms. Field and short-term laboratory studies have shown that ASS runoff can cause significant shell dissolution and perforation of heavily shelled organisms, e.g. bivalves (oyster) and gastropods [1,10,11]. However, less information is available for non or weakly calcified organisms (crustaceans, polychaetes) and for community level responses in general for extraordinarily acidified tropical estuaries [3-5]. The timeframes for exposure to ASS groundwater runoff in estuarine systems are unclear, but exposure could last for decades or hundreds of years, allowing assessment of multi-generational impacts of low pH on biological systems [3].

In addition to ASS acidification, it is possible that estuarine biota may face increasing acidification stress through present and future elevations in atmospheric CO_2_, a phenomenon affecting oceanic waters, known as ‘ocean acidification’ [10,12-15]. Because ocean acidification affects the balance of the carbonate systems, shelled organisms possessing calcium carbonate structures are expected to be especially threatened by this process. This brings to question how future OA might affect estuarine systems, and raises the importance of understanding of benthic community structuring in already acidified tropical estuarine systems. The few investigations that have looked at the effects of acidification on benthic fauna of estuaries have reported lower diversity and abundance in soft bottom communities in the acidified sites compared to reference sites [3,16,17]. However, these studies have mainly focused on the effects of ASS water inflows on epibenthic communities, without considering relationships between the pore-water pH/salinity and the faunas living within sediments. Soft-bottom infaunal organisms are expected to experience pH levels lower than those in the water column as a consequence of significant microbial and animal metabolism (including sulphide production) and poor ventilation within sediments.

Recent studies suggest that increasing acidification should have strong direct and indirect effects on marine invertebrates. These studies sometimes exclude details of the structural complexity, ecophysiological variability and genetic diversity characterizing natural communities [18]. It is however likely that communities which have evolved in acidified coastal and marine environments (such as acidified estuaries), possess species already well adapted for acidification exposure. Structural complexity should arise from taxonomic differences in physiological and behavioural adaptations, and tolerances, of low pH. As an example, crustaceans (crabs and prawns) are possibly better at tolerating reduced pH, given their ability to generally to regulate internal body fluids (with respect to most ions), compared to other taxa for which body fluids conform in ionic concentrations relative to the external environment (annelids and molluscs; see Wittman and Portner [19]). However, regulation comes at an energetic cost, and some groups are well adapted, behaviourally and physiologically, to isolate themselves from environmental perturbations. Furthermore, no studies found to show how benthic infaunal animals and communities (as opposed to epibenthic communities) of any marine system, which experience relatively stable, low pore-water pH’s, are likely to respond to atmospheric CO_2_-driven future acidification of marine waters.

The distributions of estuarine benthic faunas are well known to vary in relation to a variety of physicochemical gradients [20,21]. The main factors, however, that regulate benthic species composition and density are salinity, dissolved oxygen and substratum particle composition [20,22,23]. Among these variables, salinity fluctuations are usually overwhelming in their effect on the structure and functioning of estuarine systems [21,23-25]. Additionally, in the case of benthic infaunal communities, sediment granulometry has proven to be important [23], especially considering that estuarine sediments are extremely dynamic, comprising a variety of substrata from non-vegetated soft mud, fine and coarse sand, to vegetated saltmarsh, algal mats, seagrass beds and mangrove swamps [21,24].

Given the fact that physical stressors are likely to increase with reductions in salinity and pH of estuarine waters, we investigated whether communities in regions occupying low salinity/high acidity are characterized by relatively lowered abundance and diversity, in the case of benthic infauna communities of acidified estuarine systems. In particular, we determined variation in species abundance, species diversity and the structuring of these communities along the steep salinity and pH gradient of the Brunei estuarine system (BES, Brunei Darussalam, tropical South East Asia, Borneo). We further assessed the interaction strength for community attributes and the environmental parameters, including sediment properties.

## Results

### Pore-water characteristics

**Figure 1:**
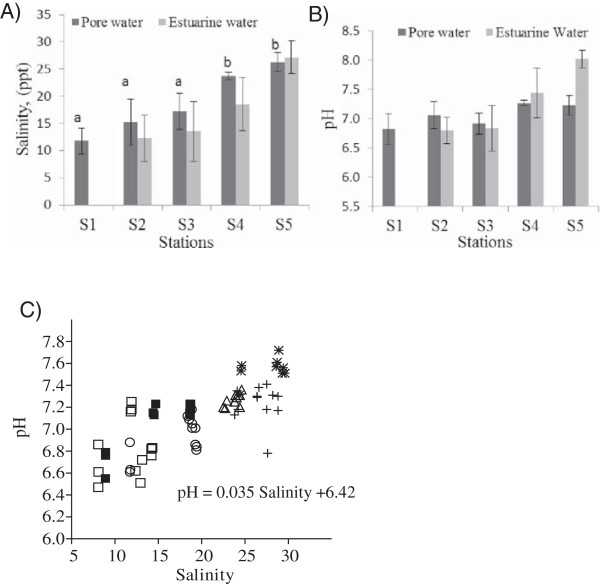
**Sediment pore-water and above-sediment estuarine water properties at the five stations along the BES.** Stations differed significantly in pore-water salinity (Wald =121.2; p <0.001) **[A]** and pH (Wald =14.2; p <0.006) **[B]**. ‘a’ and ‘b’ indicating significant difference at 5% level. The pore-water salinity and pH is correlated **[C]**. Estuarine water salinity and pH data were taken from Marshall et al. [11]; their study did not cover the site S1, hence the salinity and pH data were not available for S1.

Overall the pore-water salinity varied between 8.07 and 29.6 psu, and pH between 6.47 and 7.72. Pore-water salinity was generally greater than the above-sediment water salinity, though these were well linearly related (pore water salinity =7.62 + 0.72 overlying water salinity; r^2^ = 0.875 for means at each station, p =0.062) (Figure 1A). However, there was a poor relationship between pore-water and above-sediment estuarine water in the case of pH (pore-water pH =5.49 + 0.228 overlying water pH; r^2^ = 0.63, p =0.2), due to *in situ* sediment biotic generation of fulvic and humic acids (through heterotrophic metabolism) at stations having relatively high pH (S5 for example) (Figure 1B). Nonetheless there was an overall tendency for pore-water salinity and pH to vary as predicted (low landwards and high seawards). The relationship between pore-water salinity and pH is: pH =0.035 Salinity +6.42 (Figure 1C).

### Species composition

A total of 4174 individuals, belonging to 36 species from six taxonomic groups (Polychaeta, Copepoda, Amphipoda, Tanaidacea, Cumacea and Isopoda) were recorded in the Brunei estuarine system over the sampling period. The dominant group both in number of species (25 species) and individuals (75%) was the Polychaeta. Composition of species in different stations along the estuary clearly differed. The 10 most abundant species in the estuary, representing 93.3% of the collected benthic infauna, were *Neanthes* sp. (23.2%), *Onuphis conchylega* (9.8%), Nereididae sp.2 (9.7%), Corophiidae sp. (8.7%), Capitellidae sp.1 (8.3%), Cyclopoida sp. (7.9%), *Goniada* sp.(5.6%), Nereididae sp.3 (5.2%), *Prionospio* sp. (2.5%) and Amphipoda sp. 2 (2.44%). The dominant species also varied for different stations along the estuary: Nereididae sp.2 (43.8%) at S1, *Neanthes* sp. (35.0%) at S2, Corophiidae sp. (46.4%) at S3, *Onuphis conchylega* (18.4%) at S4 and Capitellidae sp.1 (21.1%) at S5.

**Figure 2:**
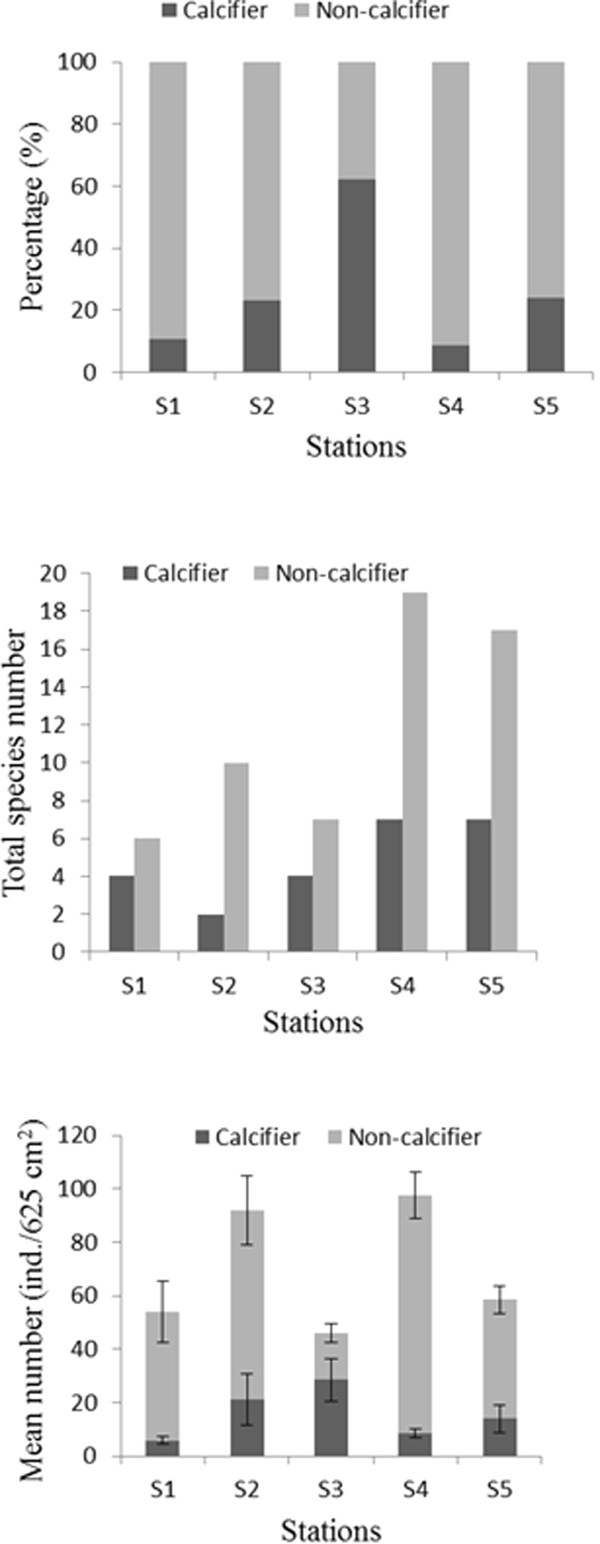
Percentage composition, species number and mean density of calcifiers and non-calcifiers in different stations along the BES.

Analyses based on the distinction of calcifiers and non-calcifiers, showed that calcifiers were highest (62.14%) in the inner low pH station (S3) and the lowest (8.80%) in the downstream station (S4) (Figure 2). In contrast, non-calcifiers were the highest (91.20%) in the downstream high pH station (S4) and the lowest (37.86%) in the acidic station (S3). Although, there was no clear spatial trend in their % composition and mean abundance (Figure 2) along estuarine pH gradient, but species number of calcifiers and non-calcifiers tended to increase towards high pH downstream stations (Figure 2). All species were included for data analysis.

### Variation of community parameters

**Figure 3:**
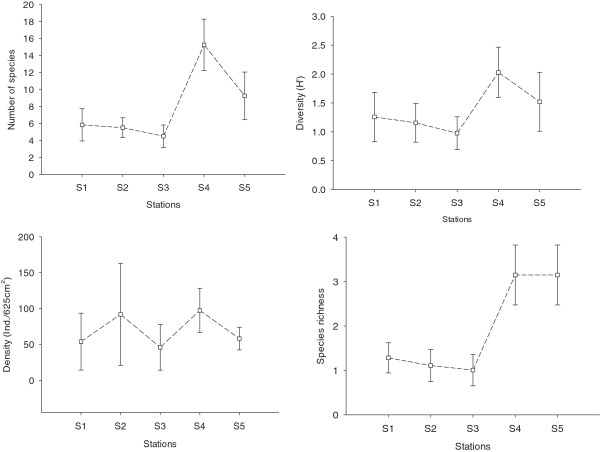
**Variation in ecological indices (number of taxa, density, species richness, diversity; mean ± SD) at different sites along the Brunei estuary.** Squares connected with a dot line represent the mean for all indices based on 12 samples at each station. Number of taxa indicates the actual number of species in each sample, and the species richness is Margalef index.

Spatial variation in the number of species, density, species richness and diversity values for each station are presented in Figure 3. Except for evenness (λ^2^ = 1.46, df =4, p =0.83), which was uniform among the stations, Kruskal-Wallis ANOVA indicated significant differences in species number (λ^2^ = 40.86, df =4, p < 0.001), density (λ^2^ = 14.72, df =4, p < 0.01), species richness (λ^2^ = 38.18, df =4, p < 0.001) and diversity (λ^2^ = 25.01, df =4, p < 0.001). The mean overall species number and density per site ranged from 4 to 15 (mean 8) and 46 to 97 (mean 69) ind.625 cm^−2^ respectively. Species diversity and richness per site ranged from 1.01 to 3.15 (mean 1.72) and 0.98 to 2.03 (mean 1.38). All the measured diversity indices were the highest at station 4 and the lowest at station 3 (Figure 3). Species diversity and richness varied among sites, with a general trend of outer sites (e.g., 4 and 5) having significantly higher values than those in inner sites (e.g., 1, 2 and 3) (*P* <0.05 for all).

### Variation of community structure

**Figure 4:**
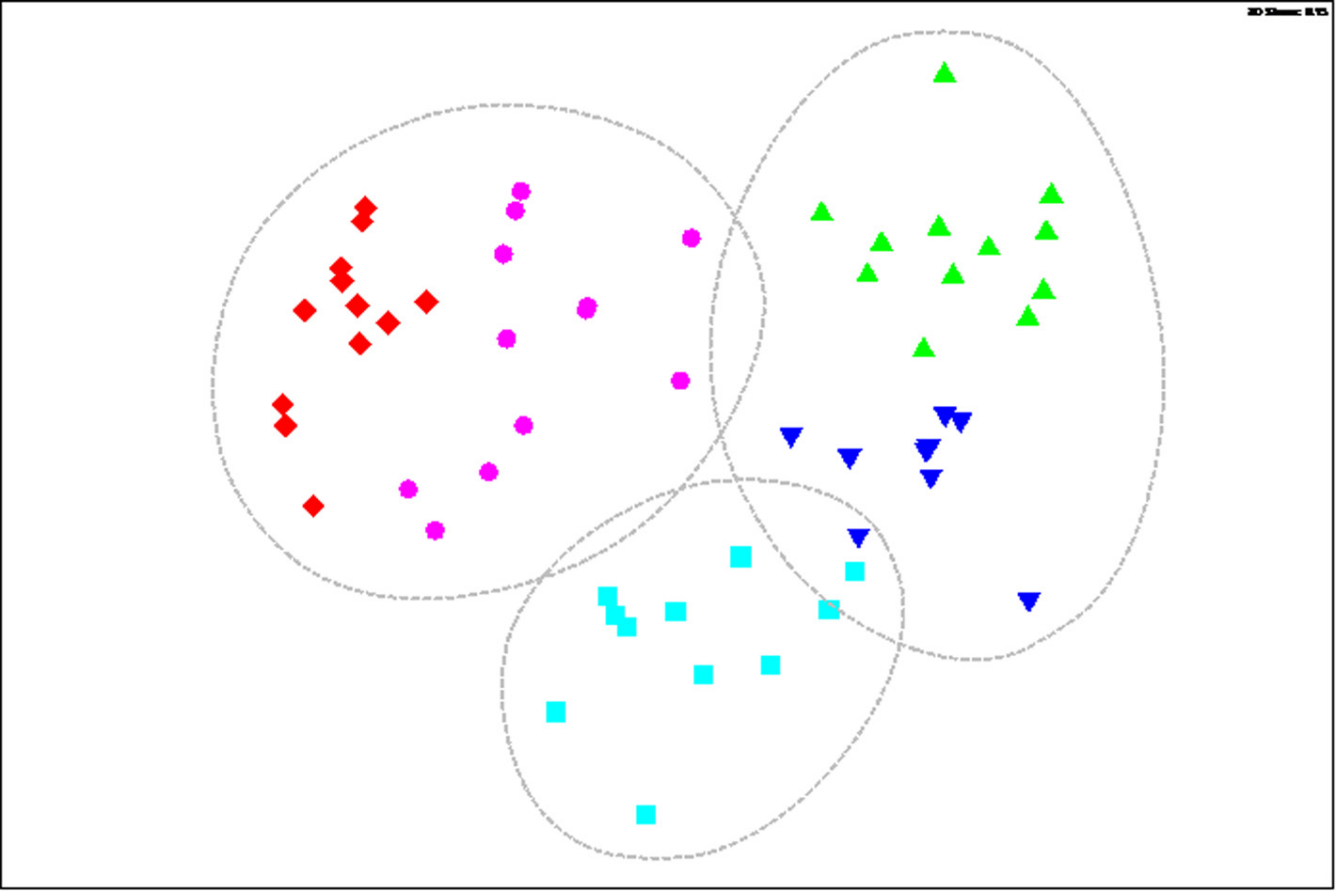
**MDS ordination constructed from Bray-Curtis similarities showing spatial variations in infuanal assemblages in the Brunei Estuary.** Data were presence/absence transformed. Key for the sites: Upward triangle: S1, Downward Triangle: S2, Square: S3, Circle: S4, Diamond = S5.

The MDS ordination grouped the infaunal assemblages into three main groups at 20% similarity level: the innermost stations S1 and S2, inner stations S3 and the outer stations S4 and S5 (Figure 4) and showed a horizontal zonation of assemblages, clearly separating outer stations from the rest of the inner stations. The low-stress value (r =0.15) in MDS showed that the community structure is well represented. Within each location, samples clustered mostly with those from the same sampling site. Samples between sites did not show a high level of similarity as indicated by the long terminal branches. The ANOSIM test confirmed the significant differences among stations (Global R = 0.977, p <0.001) (Table 1). R-statistic values for pair-wise comparisons provided by ANOSIM were used here to determine the dissimilarity between groups. Values close to 1 indicate very different composition, while values near zero show small difference. There was also a statistical evidence of temporal variability in infaunal assemblages between sampling periods (Global R = 0.548, p <0.001). SIMPER analysis of infaunal abundance data revealed that the innermost stations (S1 and S2) group was dominated by Nereididae sp.2, *Neanthes* sp. and Cylopoida sp., and the outer stations group (S4 and S5) by *Onuphis conchylega,* Capitellidae sp.1, Nereididae sp.3 and *Goniada* sp (Table 2). SIMPER revealed the average Bray-Curtis dissimilarity between locations ranged from 61.72 to 91.80%. The seaward station S4 had the highest dissimilarity values for infaunal species with most landward stations S1 (91.8%) and S2 (88.30%). The high abundance of *Onuphis conchylega* at S4 and absence of this species in S1 and S2 was a key cause of these high dissimilarities. S1 had an average dissimilarity of 82.9% with most seaward station S5, discriminating species being Nereididae sp.2 and *Neanthes* sp. The lowest dissimilarities were found between two inner stations (61.72%) and between two lower stations (64.76%), the discriminating species were Nereididae sp.3 and *Onuphis conchylega*. The dissimilarities between S1 and S3 was 84.89% and between S3 and S4 was 80.92%, and the discriminating species were Corophiidae sp. and *Onuphis conchylega.*

**Table 1:** Results of ANOSIM and pairwise tests for differences on infaunal structure between stations and sampling periods

**Global test**	**R**	**p (%)**
Between stations	0.977	0.1
Stations compared		
S1, S2	0.852	0.1
S1, S3	1	0.1
S1, S4	1	0.1
S1, S5	1	0.1
S2, S3	0.972	0.1
S2, S4	1	0.1
S2, S5	0.954	0.1
S3, S4	1	0.1
S3, S5	0.954	0.1
S4, S5	0.991	0.1

The pairwise R values give absolute measure of how separated the groups are. R >0.75: groups being well separated; R >0.5: groups overlapping but clearly different; R >0.25: groups barely separable at all. Bold types indicate separable communities.

Analyses performed on square-root transformed data.

**Table 2:** SIMPER similarity analysis of infaunal species within stations along salinity-pH gradient

**Species**	**Average abundance**	**Average similitude**	**Similitude/SD**	**Contribution %**	**Cumulative %**
Station S1					
Average similarity: 52.65					
Nereididae sp.2	4.28	23.09	2.52	43.86	43.86
*Prionospio* sp	2.35	11.02	3.01	20.94	64.80
Cyclopoida sp.	1.68	5.73	0.77	10.89	75.69
*Neanthes* sp.	1.79	5.10	0.98	9.69	85.38
*Potamilla leptochaeta*	1.84	3.37	0.60	6.40	91.77
Station S2					
Average similarity: 54.96					
*Neanthes* sp.	5.74	19.25	1.48	35.03	35.03
Cyclopoida sp.	3.83	14.39	3.23	26.19	61.21
Nereididae sp.2	2.91	9.27	1.25	16.87	78.08
Capitellidae sp.1	1.93	7.65	1.81	13.91	91.99
Station S3					
Average similarity: 59.87					
Corophiidae sp.	4.71	27.80	2.82	46.44	46.44
Capitellidae sp.1	2.17	16.54	2.57	27.63	74.07
*Neanthes* sp.	2.69	11.42	1.08	19.07	93.14
Station S4					
Average similarity: 62.53					
*Onuphis conchylega*	4.91	11.52	1.88	18.42	18.42
Nereididae sp. 3	3.97	9.76	2.92	15.61	34.03
Capitellidae sp.1	3.01	7.97	5.03	12.75	46.79
*Goniada* sp.	3.14	5.73	1.15	9.17	55.95
*Magelona* sp.	1.66	4.19	4.39	6.70	62.66
Pilargidae sp.	1.70	3.96	1.87	6.34	69.00
Maldanidae sp.	1.38	3.18	1.72	5.09	74.09
*Pholoe* sp.	1.37	2.48	1.04	3.97	78.06
*Nephtys* sp.	1.11	2.17	1.01	3.47	81.53
Corophiidae sp.	1.01	1.70	0.82	2.71	84.24
Spionidae sp.	0.89	1.48	0.83	2.36	86.60
Harpacticoida sp.	1.06	1.40	0.65	2.24	88.84
Paranthuridae sp.	1.00	1.31	0.65	2.10	90.94
Station S5					
Average similarity: 46.79					
Capitellidae sp.1	2.55	9.88	3.54	21.11	21.11
*Goniada* sp.	2.20	7.91	1.65	16.91	38.02
*Neanthes* sp.	3.05	7.14	0.71	15.26	53.27
Pilargidae sp.	1.67	5.66	1.70	12.10	65.38
*Onuphis conchylega*	1.82	4.76	0.88	10.16	75.54
Harpacticoida sp.	1.28	3.21	1.01	6.86	82.40
*Prionospio* sp	0.94	3.04	1.06	6.51	88.91
Amphipoda sp. 2	1.66	2.11	0.45	4.51	93.41

### Correlation between community parameters and environmental variables

The Spearman’s rank correlation analysis between the community parameters and the environmental variables indicated that all of the diversity indices were positively correlated with pore-water salinity and pH (p <0.05) and negatively correlated with clay and organic matter, with the exception of evenness values (p >0.05), which did not show any significant correlation with any of the environmental variables (Table 3). There was no significant correlation between community parameters and sand/silt (p >0.05).

**Table 3:** Spearman’s rank correlation coefficients (r) between environmental and community variables estimated for all species

**Variables**	**Number of species (S)**	**Density (D)**	**Richness (d)**	**Diversity (H’)**	**Evenness (J’)**
Salinity	0.648****	0.279*	0.573****	0.446***	0.010 ns
pH	0.637***	0.315*	0.562****	0.492***	0.118 ns
Sand	0.114 ns	0.018 ns	0.150 ns	0.0565 ns	0.007 ns
Silt	−0.114 ns	−0.018 ns	−0.150 ns	−0.0565 ns	−0.007 ns
Clay	−0.451***	−0.107 ns	−0.450****	−0.349**	−0.096 ns
OM	−0.570****	−0.030 ns	−0.581****	−0.390**	−0.056 ns

*p < 0.05, **p < 0.01, ***p < 0.001, ****p < 0.0001.

### Relationship between the infaunal assemblage and environmental factors

**Figure 5:**
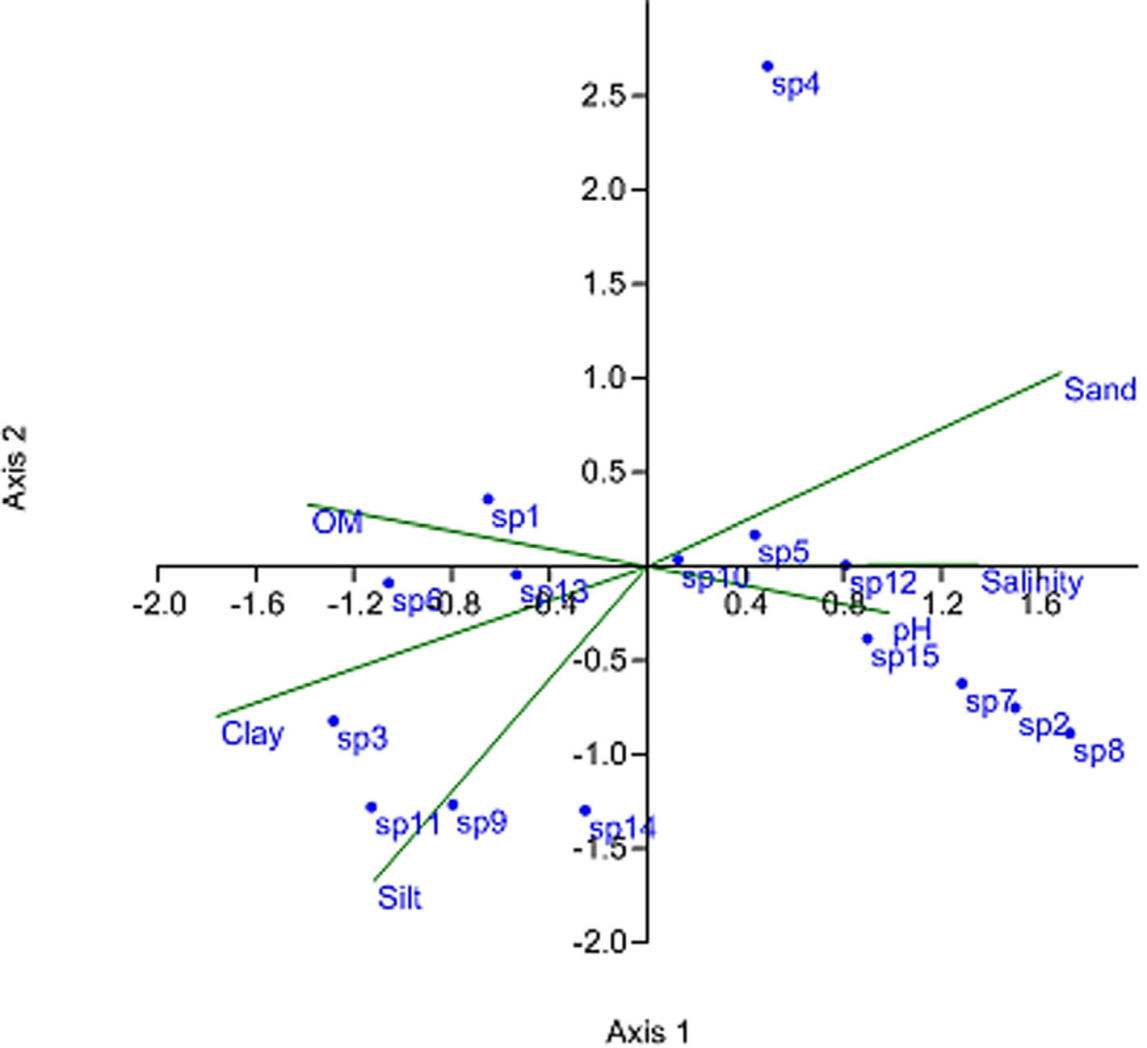
**Canonical correspondence analysis (CCA) ordination diagrams for infaunal species abundance data (occurred >1% of total abundance).** Species scores along the first and second axes in relation to environmental variables. Sediment parameters reported in Hossain et al. [26] were used. Environmental data were square root/ log transformed as necessary. The environmental variables are shown as line vectors, and the directions of which are obtained from the correlation of the variable to the axes. Species: sp1 = *Neanthes* sp; sp2 = *Onuphis conchylega*; sp3 = Nereididae sp.2; sp4 = Amphipoda sp.1; sp5 = Capitellidae sp.1; sp6 = Cyclopoida sp.; sp7 = *Goniada* sp.; sp8 = Nereididae sp. 3; sp9 = *Prionospio* sp.; sp10 = Amphipoda sp.1; sp11 = *Potamilla leptochaeta*, sp12 = Pilargidae sp.; sp13 = *Sternaspis scutata;* sp14 = Spionidae sp.; sp15 = Harpacticoida sp.

CCA for abundance of the 15 dominant species and six environmental variables produced an ordination plot in which the first two axes explain 71.31% of the variance in the species-environment relationships (Figure 5). The first axis showed highest positive correlation with salinity (r =0.83), pH (r =0.82) and percentage of sand (r =0.78), and a negative correlation with percentage of clay (r = −0.84) and organic matter (r = −0.74). This axis mainly reflected salinity and % clay, which was closely related to the location of the stations. The second axis had the strongest negative correlation for a percentage silt (r = − 91). The CCA also revealed the relationships among 15 benthic infaunal species and environmental variables. *Onuphis conchylega*, Nereididae sp. 3, *Goniada* sp. Harpacticoida sp. Pilargidae sp., and Corophiidae sp. were placed on the right side of the plot. This indicates that the distribution of these taxa at the outer stations (i.e., high salinity/pH and sandy habitat) of the estuary. *Neanthes* sp., Nereididae sp.2, Cyclopoida sp. *Prionospio* sp, *Potamilla leptochaeta*, Spionidae sp. were found on the left side of the plot (lower salinity/pH and muddy habitat) suggesting these species are mainly distributed across the inner stations. Other dominant species Capitellidae sp.1, Amphipoda sp. 2 and *Sternaspis scutata*, which were widely distributed along the estuary, fell near the origin of the plot.

## Discussion

Analysis of the sediment pore-water showed a decline of salinity and pH from sea to landwards, like the pattern reported for overlying estuarine water salinity and pH [11]. The salinity gradient in overlying water, due to the interaction of incoming sea water and outflowing of freshwater, along the estuary from inner to lower reaches is an established fact [22,23]. Ram and Zingde [27] reported that the interstitial water chlorinity is sensitive to the changes in the composition of the overlying water up to a certain depth decided by factors such as sediment type, porosity, the diffusion rate and bioturbation. However, pH variation in estuarine systems is strongly influenced by biological activity in addition to physical factors. The lowering of above sediment estuarine water pH at the landward stations of the Brunei estuary is influenced by the massive acidic freshwater outflow, but also biogenically through the production of carbonic acid from bacterial decomposition of organic matter [11]. High pH at the seaward stations results from the buffering effect of sea water [28]. Sasekumar [29] reported the acidic pH value of pore-water (varied from 6.3-7.1) from a Malayan mangrove shore and suggested that the acidity was caused by the activity of bacteria on oxidizable sulphur. The CO_2_ arising from the decomposition of organic matter and animal respiration may also lower the pH values in the sediment pore-water [30,31]. Zhai et al. [31] reported a significant outgassing of CO_2_ from Pearl River estuary which was associated with decomposition of organic pollutants by aerobic respiration. The higher pore-water salinity compared to overlying water observed in this study (Figure 1) may be attributed to evaporation of sediment surface water as the intertidal sediment at low tide was exposed to air and sunlight during sampling. However, the pore-water pH in the estuary was less variable than overlying water at the same station.

The infaunal species composition was found to vary along the BES salinity/pH gradient, though most species were euryhaline. The taxa, *Neanthes* sp*.,* Nereididae sp*.,* Capitellidae sp*., Potamilla leptochaeta, S. scutata,* Cyclopoida sp*., Leptochelia* sp.*,* extended along the length of the estuary, even tolerating salinities of 8 ppt, but showed greatest densities in the upper estuary. The stenohaline marine component was represented by the species *Onuphis conchylega, Pholoe* sp*., Syllis* sp*, Pectinaria* sp. and Calanoida sp*.,* dominated in the lower estuary. Similar patterns of species distribution are known to occur in estuaries around the world [21,22,24,28]. However, the true estuarine organisms (living below 30 ppt but not in the sea) are generally the most difficult to define [32]. O*nuphis conchylega* was most abundant in the middle reaches with abundance declining seaward and entirely absent at the upper low salinity stations, suggesting that it is a stenohaline species in this estuary.

Although there was no clear linear trend in community variables along the salinity/pH gradient as in the other estuaries [22,33], species richness, density, diversity and number of taxa in the benthic infauna were all positively related to salinity (*p* <0.05 for all). This is due to a reduction in stenohaline species at the inner stations [23,34] and lessened substrate diversity following a progression from sand at the lower reaches to mud at the upper reaches. The relationship between environmental variables and measured diversity indices explained this pattern as significant positive correlations were found for pore-water salinity, pH and % sand, while a negative correlation was found between % clay and organic matter (p <0.05) (Table 3). For infaunal macroinvertebrates, sediment grain size has frequently been reported as a crucial factor in determining the structure of many benthic communities [22,35-40].

Most studies suggest that calcifying animals should be more affected by low pH waters than non-calcifiers [3-5,15,41,42]. However, our abundance data showed no different trend in distribution of calcifiers and non-calcifiers along the pH gradient. The high abundance of few calcifiers in inner low pH stations (Cyclopoida sp. in station S2 and the amphipod Corophiidae sp. in S3) essentially differs from the predicted trend [15,43]. The wide-ranging abundance of infaunal calcifiers may relate to (1) the reduced gradient in the pore-water in the BES, (2) adaptations to endure lowered pHs, (3) biological interactions where the predators are less tolerant of slightly higher acidic conditions. The high number of tolerant species in the acidic areas may also be due to the absence of metal sensitive predators [16]. Furthermore, organisms in coastal and estuarine waters have been experiencing low pH for several hundreds of years and consequently have potentially had time to adapt to low pH [3,42]. Several groups of invertebrates are physiologically resistant to low pH; for example, crabs can tolerate low pH through their physiological acid–base regulation [3,19,44]. Recent studies suggest that calcifiers are as sensitive to acidification as heavily calcified animals [3,15,41,42]. The findings of infaunal microcrustaceans (amphipods, isopods, cumaceans, tanaids, and copepods) are consistent with these previous findings suggesting a size-related difference in acidic vulnerability [5,15,42].

Many estuarine benthic studies [e.g., [22,33,45,46] have shown that community variables are principally correlated with water salinity, DO and sediment grain size, and this has been confirmed in the present study. Relatively few studies have explored the effect of pH on estuarine infaunal communities [16,17]. However, earlier work [16,17] emphasized the relationships between water column parameters and benthic infauna, rather than focusing on the sediment pore-water, with which these organisms directly interact. Therefore, this the first study investigating the effect of pore-water pH on infaunal community structuring in a tropical estuary. Consequently, the unavailability of data constrains comparison. Nonetheless, the community level responses observed here are consistent with observations on the effects of acidification for other aquatic (marine) systems, including (1) a decrease in total community diversity [43], (2) a high abundance of crustaceans [15], and (3) a shift in the community composition [47].

In conclusion, this study shows that sediment particle size probably overrides effects of the above sediment water, and that the sediment (pore) water pH did not vary as much as the above water pH (primarily due to biogenic acidification of the pore-water). The implication of the latter is that coastal infaunal communities may be less affected by water pH variations caused by various ways including elevation in atmospheric CO_2_ than what has been described for epibenthic communities.

## Materials and methods

### Study site

**Figure 6:**
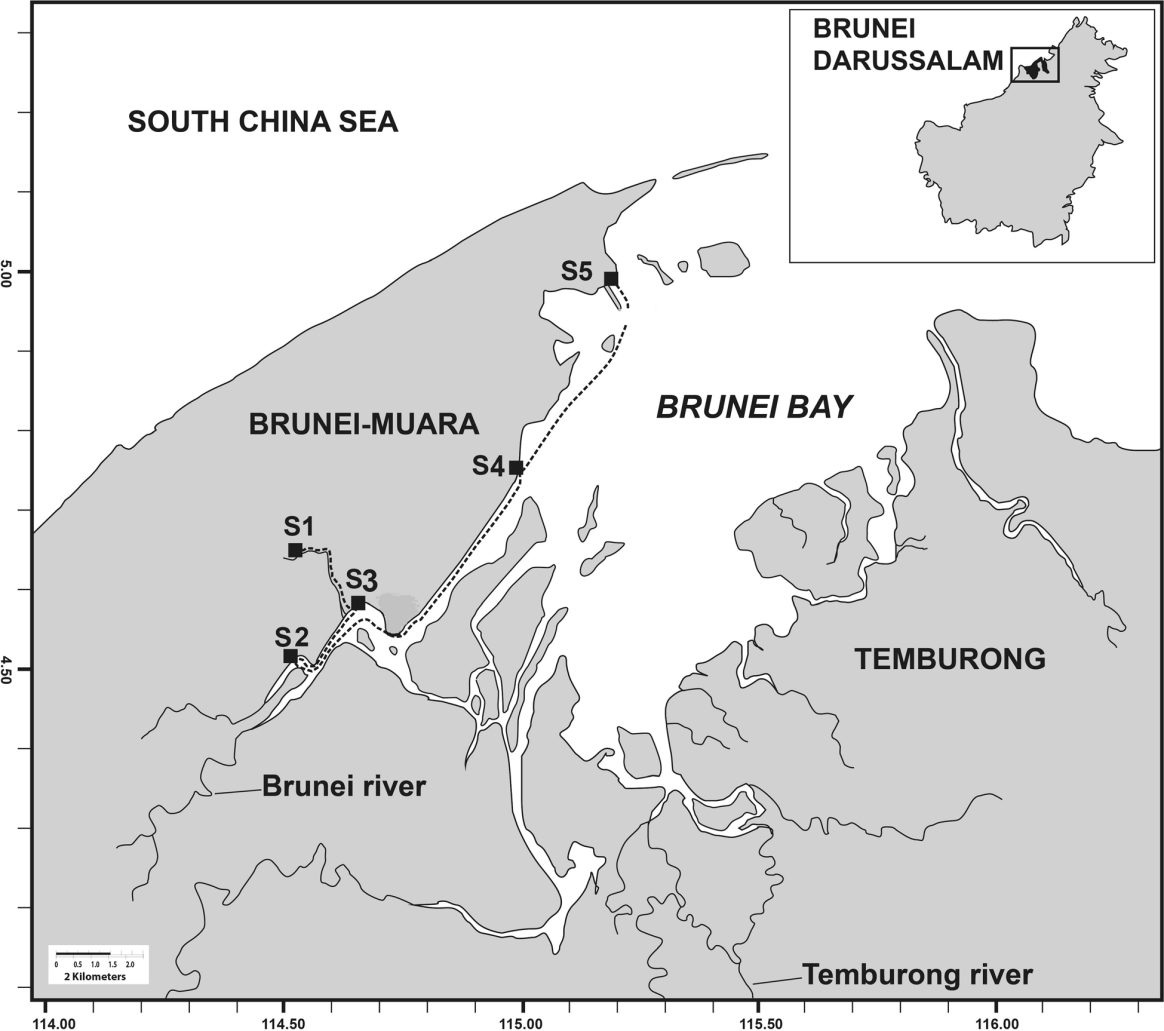
**Map of the study area indicating sampling sites (S1, Kiulap; S2, Damuan; S3, Bandar; S4, Sg Besar and S5, Serasa Bay).** The mean estuarine water pH at stations along the estuary: S2 (7.06), S3 (6.92), S4 (7.27), S5 (7.23). Estuarine water salinity and pH data were taken from Marshall et al. [11].

The BES (Figure 6) constitutes the Inner Brunei Bay and three major river systems (Sungai Limbang, Sungai Temburong and Sungai Brunei) flanked by the South China Sea, Brunei and Malaysia in North-western Borneo (1° 00' 00" N and 114° 00' 00" E). It occupies an area of 1380 km^2^. The equatorial tropical climate in the region ensures high rainfall and temperature conditions throughout the year, with regular freshwater inflow into the shallow (<5 m depth) and well-mixed system [26]. The shoreline is fringed predominantly (75%) by *Rhizophora* mangrove forests [48]. The tides are mainly diurnal with semi-diurnal on a few days, and the daily tidal amplitude ranges from about to 0.9 m to 2.0 m at the estuarine mouth. Although the agricultural and industrial sectors in the region are under-developed, the estuary is subject to significant solid waste pollution, including treated and untreated sewage and domestic waste from traditional water villages, urban developments (Bandar Seri Begawan, Kuilap and Gadong [11,49].

The estuary is naturally acidic, primarily due to eutrophication, heterotrophic metabolism and associated acid sulphate groundwater seeps [11]. Consequently, a steep cline in estuarine water salinity and pH extends across the system; low salinity/pH landwards and high salinity/pH seawards. Five study stations were established along the northern edge of the Inner Brunei Bay and Sungai Brunei estuary (Figure 6).

### Pore-water salinity and pH determination

At each station, *in situ* pH and salinity of low-tide sediment pore-water was determined. A pit of 20 cm in depth was dug using spade, and pH and salinity of the water seep was measured using Hach HQ series portable salinity meters (CDC 401–01, USA) and Mettler Toledo pH probe (Type 1120, Germany) calibrated with Mettler Toledo SRM NIST precision buffer solution (using pH 4, 7, and 10 standards). In each field visit at least five stabilized readings were taken from each sampling spot. Details of methodology and results for sediment properties (% of sand, silt, clay and organic matter) were reported in the earlier study [26].

### Infaunal sampling and determination

Macrobenthic infauna were sampled at five stations in Brunei estuary (Figure 6) on four occasions from July 2011 to June 2012. Stations 1 and 2, in the upper reaches of the estuary, had a muddy black bottom which was anoxic and with the smell of H_2_S. Station 3, located in the upper reaches but with sandy mud bottom and station 4 and 5, at the lower end of the estuary, had a sandy mud bottom. Three replicate samples were taken at each station from mid-intertidal area on each date. One additional core was taken from each station for determination of sediment physical characteristics. Sediment samples were excavated from an area of 25 cm × 25 cm with a depth of 20 cm by using a spade during low tide. Excavated sediment samples were put into polyethylene bag and carried to the laboratory where sediment samples were washed through 0.5 mm mesh sieve with tap water. Detritus and organisms retained by the sieve were stained in dilute Rose Bengal and fixed in 5% formalin for one day. The formalin was later washed out and specimens were preserved in 70% ethanol. Individuals were separated from the sediment and detritus under a dissecting microscope and preserved and kept in small vials of ethanol for future identification. Animals were identified to the lowest known taxonomic level using available identification sources [e.g., [48-54]. Within each family, fauna were distinguished as ‘morphospecies’ [55]. The taxonomic status was checked and updated using the web portal WORMS (http://www.marinespecies.org). Densities of taxa (number of individuals/625 cm^2^ surface area) were determined by counting all organisms in samples. Only the dominant taxa (Polychaeta and Crustacea) were used in the analysis and we assumed interacted closely with the sediment pore-water. All the specimens are deposited in the Biology Department museum, Universiti Brunei Darussalam, Brunei.

### Statistical analysis

Univariate statistical analyses were undertaken to determine for each station the number of species (taxa), average density (D), Margalef’s species richness (d), Pielou’s evenness (J’), Shannon-Weiner diversity (H’, loge base). On the assumption that the data were not normally distributed, nonparametric Kruskal-Wallis ANOVA and Mann–Whitney pair-wise comparison tests were performed to assess significant differences in the diversity indices among sites. These analyses were run using PAST [56]. Multivariate statistics were used to investigate variations in the structure of the infaunal community throughout the study period. A Bray-Curtis similarity matrix was computed using abundances of fauna, from which cluster analysis and the non-metric multidimensional scaling (MDS) ordination plot was generated (based on presence/absence transformed data) to visualize the patterns in the spatial distribution using PRIMER V.6 [57,58]. The programme was also used to compute a two-way analysis of similarity (ANOSIM), to determine significant spatial variation in the faunal communities. Taxa making the highest contribution to the differences were detected using SIMPER (PRIMER).

Spearman’s rank correlations were calculated to show the relationship between univariate measures and environmental parameters. Sediment parameters reported in Hossain et al. [26] were used in order to provide a basis for interpretation of the biological variables. Associations between assemblage patterns and environmental variables were quantified via canonical correspondence analysis (CCA), a nonlinear eigenvector ordination technique related to CA but which constrains the axes to be linear combinations of the measured environmental variables (run in PAST).

## Competing interests

The authors declare that they have no competing interests.

## Authors’ contributions

This is Postgraduate research (Ph.D.) work of the first author. DJM supervised the work and contributed to this study significantly, and were involved at different times: sampling designing, data analyzing and improving the English language. Both authors read and approved the final manuscript.
